# Porcine SCD1 Regulates Lipid Droplet Number via CLSTN3B in PK15 Cells

**DOI:** 10.3390/ani15111663

**Published:** 2025-06-04

**Authors:** Wenzhe Bai, Qianhai Fang, Yanzhen Bi, Rui Wang, Ke Xu, Ao Zhou, Hao Gu, Hongbo Chen

**Affiliations:** 1 Laboratory of Genetic Breeding, Reproduction and Precision Livestock Farming, Hubei Provincial Center of Technology Innovation for Domestic Animal Breeding, School of Animal Science and Nutritional Engineering, Wuhan Polytechnic University, Wuhan 430023, China; baiwenzhe1@126.com (W.B.); zjgfeixiang@163.com (R.W.); 22113033@whpu.edu.cn (K.X.); aoqiu@whpu.edu.cn (A.Z.); 2 Key Laboratory of Animal Embryo Engineering and Molecular Breeding of Hubei Province, Wuhan 430064, China; fangqianhai_98@163.com (Q.F.); sukerbyz@126.com (Y.B.)

**Keywords:** pig, *SCD1*, *CLSTN3B*, lipid droplet, meat quality

## Abstract

Fat deposition plays a key role in determining porcine meat quality traits, with lipid droplets serving as critical organelles for lipid storage in adipose tissue. Inhibiting lipid droplet biogenesis disrupts the lipid storage capacity of adipocytes. The Stearoyl-CoA Desaturase (*SCD*) family is crucial in regulating polyunsaturated fatty acid/monounsaturated fatty acid (PUFA/MUFA) composition, while its role in lipid droplet formation remains unclear. This study employed CRISPR/Cas9 to create SCD1-deficient porcine renal epithelial cells (PK15), enabling an investigation into SCD1’s role in fatty acid composition and lipid droplet regulation. RNA-seq analysis was conducted to elucidate the mechanisms underlying SCD1’s impact on lipid droplet numbers. Results showed that SCD1 deletion significantly decreased triacylglycerols (TAG) content, altered fatty acid composition, and decreased lipid droplet numbers. Conversely, SCD1 overexpression increased lipid droplet numbers, confirming SCD1’s role in regulating lipid droplet abundance. RNA-seq analysis revealed that SCD1 regulates lipid metabolism via Calsyntenin 3β (*CLSTN3B*). Experimental validation confirmed the SCD1-CLSTN3B regulation of lipid droplet numbers. In summary, we discovered the role of SCD1 in regulating the number of lipid droplets, highlighting its potential impact on lipid metabolism and adipocyte function in pigs.

## Simple Summary

Fat deposition is a critical determinant of pork quality traits, with lipid droplets serving as essential organelles for lipid storage in adipose tissue. However, the role of *SCD1* in lipid droplet biogenesis remains unclear. In this study, we utilized CRISPR/Cas9 to generate SCD1-deficient cells and found that SCD1 regulates both fatty acid composition and lipid droplet number. RNA-seq analysis further revealed an association between SCD1 and CLSTN3B, and experimental validation demonstrated that SCD1 modulates lipid droplet number via targeting CLSTN3B.

## 1. Introduction

The intramuscular fat (IMF) content is a crucial indicator of pork quality and has attracted significant research attention [1]. Triacylglycerols (TAG) and phospholipids (PL), as the main components of IMF, are key contributors to its deposition. Lipid droplets (LD) serve as the primary storage structures for neutral lipids, including free fatty acids (FAs), TAG, and cholesteryl esters (CE), and are enclosed by a phospholipid monolayer. This suggests that LD accumulation is closely associated with IMF deposition, highlighting its essential role in fat storage and meat quality formation [2,3]. Among them, fatty acid composition also has an important influence on pork quality, flavor, and nutrition [4]. The level of monounsaturated fatty acids (MUFA) affects meat flavor and tenderness [5]. The increase in IMF content is positively correlated with that of saturated fatty acid (SFA) and MUFA, and negatively correlated with that of polyunsaturated fatty acid (PUFA) [6,7].

Stearoyl-CoA Desaturase 1 (*SCD1*) is a key enzyme in FA biosynthesis that catalyzes the conversion of SFA to MUFA [8]. The conversion of palmitic (16:0) and stearic (18:0) acids to the MUFA palmitoleic (C16:1) and oleic (C18:1) acids is catalyzed by SCD1, whose specific catalytic products, palmitoleic (C16:1) and oleic (C18:1) acids, are important control targets for fatty acid metabolism [9]. These FAs represent the major components of adipocyte TAG and PL, playing important roles in lipid droplet expansion [10,11]. In the subcutaneous white adipose tissue of mice, SCD1 is activated by cold exposure and promotes TAG mobilization [12]. In bovine subcutaneous and visceral fat, SCD1 is highly expressed. SCD1 expression in the stromal vascular fraction cells of cattle promotes lipid droplet accumulation [13]. Pharmacological inhibition of SCD1 expression affects cell viability and impairs TAG accumulation and LD formation via lipolytic and lipophagic pathways [14]. However, most of the current studies on *SCD1* are about fat deposition, and there are few studies on the mechanisms by which *SCD1* regulates lipid droplet biogenesis.

Calsyntenin 3β (*CLSTN3B*) is an isoform distinct from CLSTN3 and consists of three exons. In brown adipose tissue, its first exon resides within intron 16 of *CLSTN3*, while the remaining two overlap with exons 17 and 18 of *CLSTN3*. Notably, *CLSTN3B* has been identified as a key regulator of sympathetic innervation in both brown and beige adipose tissues. It functions through the S100B axis to facilitate neurotransmitter transmission. The regulation of sympathetic innervation by CLSTN3B is critical for thermogenesis, as sympathetic activation triggers the β-adrenergic receptor pathway in adipocytes, thereby promoting lipolysis and UCP1-mediated non-shivering thermogenesis. Furthermore, by strengthening sympathetic signaling, CLSTN3B enhances adipocyte adaptability to environmental stimuli, such as cold exposure, ultimately driving energy expenditure. These findings suggest that CLSTN3B may play a pivotal role in maintaining thermoregulation and energy homeostasis [15]. Emerging evidence further indicates that CLSTN3B modulates lipid droplet morphology, keeping adipocytes in a multilocular rather than unilocular state. The presence of multiple small lipid droplets is commonly associated with heightened lipolysis and enhanced lipid oxidation, promoting efficient fat mobilization for energy. This structural adaptation is particularly evident in brown and beige adipocytes, underscoring the potential role of CLSTN3B in cold adaptation and high-energy-demanding conditions [16]. Additionally, CLSTN3B plays a crucial role in adipocyte lipid droplet maturation. It employs arginine-rich segments to facilitate extensive contact and the formation of hemifusion-like structures between ER and LD membranes, enabling phospholipid diffusion from the ER to LDs during LD expansion [17].

It has been reported that the porcine renal epithelial cell (PK15) is a suitable model in the fat deposition research [18,19]. This study used the CRISPR/Cas9 system with a single sgRNA to generate SCD1 knockout cell lines in immortalized porcine renal epithelial cells (PK15). This allowed investigation of SCD1’s regulatory role in lipid metabolism and lipid droplet biogenesis, providing an insight for the genetic improvement of pork quality.

## 2. Materials and Methods

### 2.1. Cell Lines

The PK15 cells were maintained in complete DMEM (Gibco, Beijing, China) supplemented with 10% fetal bovine serum (FBS, Hamilton, New Zealand), 100 IU/mL penicillin, and 100 μg/mL streptomycin (Gibco, Beijing, China) with media changes every two days. The cells were cultured in an incubator at 39 °C with 5% CO_2_ and 95% air. The monoclonal cell line was expanded and passaged for more than ten generations before being identified to test its genetic stability. When the cells reached 80% confluence, they were collected for relevant experiments. The PK15 cell line was provided by the Institute of Animal Husbandry and Veterinary Medicine, Hubei Academy of Agricultural Sciences.

### 2.2. Cell Transfection and Monoclonal Acquisition

The first exon of the porcine SCD1 gene (NCBI No. NC_010456.5) was selected as the target region. The online target site prediction tool (http://crispor.tefor.net accessed on 4 May 2023) was used to design sgRNA oligonucleotide sequences at exon 1 of the gene. The sgRNA was synthesized by Sangon Biotech (Shanghai, China). The sgRNA oligonucleotide sequences are provided in Supplementary Table S1. The CDS sequence of the SCD1 gene was amplified using specific primers (Table S1) and cloned into the pcDNA3.1 vector to construct the SCD1 overexpression plasmid. The pcDNA3.1-3*Flag-CLSTN3B vector was synthesized by AuGCT (Beijing, China). PK15 cells were transfected with the successfully constructed PX459-Cas9/sgRNA (pcDNA3.1-SCD1-6*His/pcDNA3.1-3*Flag-CLSTN3B) plasmid with PX459-Cas9 (pcDNA3.1) empty load as a negative control as follows: cells were transfected using electroporation at 220 V/cm, 3 ms, one pulse (ECM2001 Elector Cell Manipulator, BTX, Holliston, MA, USA). The cells were transfected with 5 μg of the recombinant plasmid in 100 μL of the system (1 × 10^6^ cells), and then screened with 12% FBS/DMEM containing 2 μg/mL of Puromycin (1 μg/mL of G418 for 5 days) for 48 h. The screened cells were allowed to proliferate into clusters, and the clones were picked up and expanded. After the single cells were screened and proliferated into cell clusters, single clones were picked and expanded in culture. The selected single-cell clones were expanded and identified. Genomic DNA was extracted for PCR analysis, and the clone with a 4 bp (AGAC) deletion was selected for subsequent experimental validation and designated as the SCD1^−/−^ cell line. To prevent the possibility of micro-deletion repair, the monoclonal cell line was further expanded and passaged for more than ten generations, followed by re-identification to assess genetic stability. The electrophoresis and sequencing results remained consistent with the initial findings, indicating that the mutation was stably inherited and suitable for further studies. Subsequently, off-target analysis was performed on the SCD1-deficient cell line to ensure the specificity of the gene editing.

### 2.3. Real-Time Quantitative PCR

Total RNA was extracted from cells using the TransZol reagent from Ambion (Thermo, Shanghai, China). After quality assessment, the reverse transcription system was prepared according to the instructions for the HIScript^®^ II QRT SuperMix kit (Vazyme, Nanjing, China). The reaction system was prepared using the ChamQ Universal SYBR qPCR Master Mix (Vazyme, Nanjing, China), following the manufacturer’s instructions. Subsequent qRT-PCR analysis was performed on the Applied Biosystems PCR system (Thermo, Shanghai, China). All reagents were products of Vazyme (Vazyme, Nanjing, China). GAPDH was used as the housekeeping gene for normalization [20], and mRNA expression was calculated using the 2^−ΔΔCt^ method. The primers used for the qRT-PCR reactions are listed in Table S1.

### 2.4. Western Blotting

Cells and tissues were lysed in RIPA (Beyotime, Shanghai, China) lysis buffer containing 1% protease inhibitor (Beyotime, Shanghai, China) for 30 min at 4 °C. The cell lysates were centrifuged at 13,500× *g* for 10 min at 4 °C, and the supernatant was collected. Subsequently, 5× SDS-PAGE sample buffer was added to the supernatant, and the mixture was boiled at 95 °C for 10 min to denature the proteins. Proteins were separated by SDS-PAGE using a two-step voltage protocol: 80 V for 30 min followed by 120 V for 1 h and 30 min. After electrophoresis, the proteins were transferred to a PVDF membrane at a constant current of 200 mA for 1 h and 10 min. The membranes were blocked in 5% skimmed milk (BioSharp, Hefei, China) for 2 h at room temperature. After three washes with Tris-buffered saline containing 0.1% Tween-20 (TBST), the membranes were incubated overnight at 4 °C with the appropriate primary antibody (SCD1, Abcam, ab39969, 1:500; CLSTN3, Proteintech, 13302-1-AP, 1:500; β-tubulin, Proteintech, 1:5000, 10068-1-AP; Anti-His, ABclonal, AE086, 1:1000; Anti-FLAG, Proteintech, 66008-4-lg, 1:2000; Anti-Rabbit, Proteintech, 1:5000, SA00001-2; Anti-Mouse, Proteintech, 1:5000, SA00001-1). The membranes were washed three times with TBST and then incubated with secondary antibodies at room temperature for 1.5 h. An ECL chemiluminescence reagent (Beyotime, Shanghai, China) was used to develop the protein sign. The imaging was performed using the FUSION FX EDGE/DBT imaging system (Vilber, Beijing, China), followed by quantitative analysis using ImageJ v1.8.0.

### 2.5. Triglyceride Content Test

The cells were inoculated into 6-well cell culture plates according to 1 × 10^5^ cells (three replicates for each sample). After the cell density reached 90–95%, the medium was removed and rinsed with PBS 2–3 times. The following steps were performed with the instructions of Tissue Cell Glycerol Assay Kit (Applygen, Beijing, China). Subsequently, detection was performed using the Victor X5 microplate reader (PerkinElmer, Shanghai, China).

### 2.6. Total Fatty Acid Content Assay

A total of 6 × 10^5^ cells were inoculated in 10 cm cell culture dishes (three replicates per sample). After the cells were confluent to 90–95%, the medium was removed, and the cells were rinsed with PBS 2–3 times; the appropriate amount of PBS was added, and the adherent cells were gently scraped with a sterile spatula; the cell suspension was collected, and the culture medium was removed by centrifugation at 4 °C, 2250× *g* for 5 min. After washing the cell samples 2–3 times with PBS, the cells were collected into centrifuge tubes and placed in a 4 °C centrifuge. The samples were centrifuged at 2250× *g* for 5 min per spin. The supernatant was discarded, and the cell pellets were quenched in liquid nitrogen to inhibit intracellular metabolic activity. The centrifuge tubes were then placed at room temperature for freeze-drying treatment. Once the cell pellets were completely dried, equal amounts of cells were accurately weighed and subjected to quantitative analysis using an AB Sciex QTRAP^®^ 6500+ mass spectrometer (Sciex, Shanghai, China).

### 2.7. Bodipy Staining Flow Cytometry Assay

When the cell confluence reached 90%, a 2 μmol/L Bodipy (Amgicam, Wuhan, China) staining solution was prepared in PBS. The cells were rinsed three times with PBS to remove residual medium and serum. Each well was incubated with 1 mL of the 2 μmol/L Bodipy solution at 37 °C for 15 min in the dark, followed by three quick rinses with PBS. Cells were digested with trypsin to obtain a single-cell suspension. Digestion was terminated, and cells were collected in 2 mL tubes and centrifuged at 1450× *g* for 3 min. The supernatant was discarded, the cells were resuspended in PBS and centrifuged, and the cells were rinsed twice. The cells were resuspended, filtered through a 40 μm mesh filter into a 50 mL tube, and the filtrate was transferred to a 2 mL tube, then centrifuged at 1700× *g* for 3 min. The supernatant was carefully removed, and the cells were resuspended in 300 μL of 1× flow cytometry buffer. The samples were analyzed for fluorescence intensity using the CytoFLEX flow cytometer (Beckman Coulter, Brea, CA, USA).

### 2.8. Bodipy Staining Confocal Imaging

Autoclaved coverslips were placed in a 35 mm cell culture dish and the cell suspension was added to the dish. The cells were made into crawls when they grew to the appropriate confluence. Add 1 mL of 4% paraformaldehyde to the culture dish and fix the samples at room temperature for 30 min. After fixation, remove the 4% paraformaldehyde and wash the samples three times with 1 mL of PBS, each for 3 min. Add 1 mL of 2 μmol/L Bodipy staining solution prepared with PBS to the culture dish and incubate at 37 °C for 15 min. One drop of anti-fluorescence quencher (Beyotime, Shanghai, China) was placed on the slide and the coverslip was placed upside down on the coverslip for imaging with a confocal microscope, imaging was performed using the NIKON Eclipse Ti confocal microscope (NIKON, Tokyo, Japan).

### 2.9. Oil Red O Stain

When the cell confluence reached 90%, the cells were stained using the Modified Oil Red O Staining Kit (Beyotime, Shanghai, China), and the cell culture was discarded and washed three times with PBS, followed by the addition of 4% paraformaldehyde solution for 30 min of fixation. The paraformaldehyde solution was discarded and washed three times with PBS for 2 min at each time. Modified Oil Red O staining solution was added to stain for 10 min, then the staining solution was carefully aspirated and PBS was added, then it was slowly aspirated and the operation was repeated twice. Hematoxylin staining solution (Solebo, Beijing, China) was added to stain for 1 min, with the subsequent aspiration of the staining solution, before PBS was added and it was washed twice. An amount of 1 mL of PBS was added to cover the cells and the cells were imaged with an ECLIPSE Ts2 fluorescent inverted microscope (NIKON, Japan).

### 2.10. RNA-Seq Analysis

When the cells reached 80% confluence, total RNA was extracted using Trizol. The RNA concentration and quality were then assessed using the Qubit^®^ RNA Analysis Kit (Life Technologies, Waltham, MA, USA) and the LabChip GXII Touch HT Nucleic Acid Analyzer (PerkinElmer, Waltham, MA, USA). RNA samples that passed quality control were used to construct sequencing libraries with the KAPA™ Single-Stranded RNA Library Preparation Kit (Illumina, San Diego, CA, USA). Sequencing was then performed on the Illumina HiSeq X platform to generate raw data. The raw data were processed using Illunima HiSeq^TM^2000 and Fastp V0.20 to remove low-quality reads and adapters, resulting in high-quality sequences (Clean Reads). Clean Reads were aligned to the reference genome (GCF_000003025.6_Sscrofa11.1_genomic.fna.gz) using Hisat2 software v2.1.0 to determine their genomic positions and identify sample-specific sequence features. Gene expression levels were analyzed using DESeq2 software v1.30.0, with a screening threshold of *p* < 0.05 and |log_2_FC (Fold Change)| > 1 to identify differentially expressed genes under different treatments.

### 2.11. Immunofluorescence

The pcDNA3.1-3*Flag-CLSTN3B overexpression vector was transfected into WT cells, followed by immunofluorescence assay. Cells were pre-seeded into cell culture dishes with coverslips and rinsed once with PBS at room temperature. They were fixed with 4% paraformaldehyde in PBS for 10 min at room temperature, followed by three washes with ice-cold PBS. The samples were permeabilized with 100 μM digitonin (Beyotime, Shanghai, China) for 10 min at room temperature, then rinsed three times with PBS for 5 min each. Blocking was performed using 5% goat serum (Solebo, Beijing, China) for 30 min at 37 °C. The cells were then incubated overnight at 4 °C with the primary antibody diluted in PBS (SCD1, Abcam, ab39969, 1:200; Anti-FLAG, ABclonal, AE005, 1:300; Cy3, ABclonal, AS007, 1:300; ABflo-488, ABclonal, AS037, 1:300). After incubation, the cells were washed three times with PBS for 5 min each and incubated with the secondary antibody diluted in 1% BSA in PBS for 1 h at room temperature. This was followed by three additional PBS washes for 5 min each. Coverslips were mounted onto microscope slides using an anti-fade sealing solution containing DAPI (Beyotime, Shanghai, China). The imaging was performed using the NIKON Eclipse Ti confocal microscope (NIKON, Japan).

### 2.12. Statistical Analysis

In GraphPad Prism 9.5.1, statistical significance between the control and treatment groups was analyzed using Student’s *t*-test. *p*-value < 0.05 was considered statistically significant. * *p* < 0.05; ** *p* < 0.01; and ns = not significant. All data are presented as the mean ± standard error of the mean (SEM) of independent biological replicates. Bodipy staining was performed in six independent biological replicates (n = 6). Oil Red O staining and RNA-seq experiments were performed in triplicates (n = 3 biological independent samples). Western blot and TAG content analysis represent data from multiple independent replicates showing similar results.

## 3. Results

### 3.1. SCD1 Deficiency Regulates Lipid Metabolism in PK15 Cells

**Figure 1:**
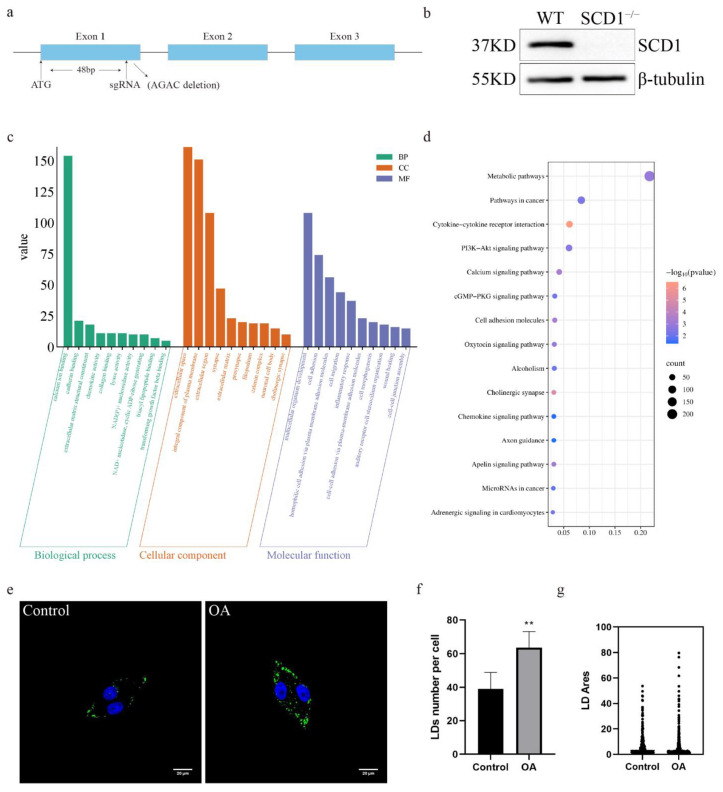
SCD1-Mediated Regulation of Lipid Metabolism in PK15 Cells: (**a**) Schematic representation of SCD1 knockdown site; (**b**) SCD1 protein expression level assay; (**c**) WT vs. SCD1^−/−^ GO pathway analysis; (**d**) WT vs. SCD1^−/−^ KEGG pathway analysis; (**e**) Oleic Acid Induces Lipid Accumulation in PK15 Cells; (**f**) imageJ calculates the number of lipid droplets in graph (**e**); (**g**) imageJ calculates the area of lipid droplets in graph (**e**). ** *p* < 0.01.

To investigate the function and role of SCD1 in pigs, exon 1 of the *SCD1* gene was targeted for knockout using a single sgRNA (Figure 1a). We used a CRISPR/Cas9 system with a single sgRNA targeting exon 1 of the *SCD1* gene. This resulted in a 4 bp deletion (AGAC) within the coding region (Figure S1a), leading to a frameshift mutation and the introduction of a premature stop codon (Figure S1b), which disrupts the normal function of the SCD1 protein. Subsequently, we performed off-target analysis of the sgRNA, and the results showed that no off-target effects occurred (Figures S2a–d and S3a–o). The mRNA and protein levels of SCD1-knockout cells were analyzed, as the level of microdeletion SCD1 mRNA remained unchanged (Figure S1c), Western blot results showed that the 37 kDa SCD1 protein band was not detected in the edited cells, and no new lower molecular weight bands were observed (Figure 1b and Figure S1d). This indicates that the mutation caused translation termination or protein degradation, preventing the formation of a stable protein structure. We performed transcriptome sequencing (RNA-seq) on WT (Wild type) and SCD1^−/−^ cells. KEGG enrichment analysis revealed that the deletion of SCD1 in PK15 cells resulted in significant changes in several biological processes, including triacyl lipopeptide binding and NAD(P)+ nucleosidase activity, as well as the molecular function of cell adhesion (Figure 1c). GO pathway enrichment analysis showed significant enrichment of pathways such as Metabolic pathways and PI3K-Akt signaling pathway and Apelin signaling pathway (Figure 1d), both of which are known to play important roles in lipid accumulation and adipose tissue remodeling. To further assess lipid storage capacity, PK15 cells were stimulated with oleic acid, resulting in a pronounced increase in lipid droplet accumulation (Figure 1e). Compared to the control group, oleic acid treatment significantly elevated both the number and size of lipid droplets (Figure 1f–g), suggesting an enhanced lipid storage potential.

### 3.2. SCD1 Knockout Affects Fatty Acid Composition and Downregulates LD Number

**Figure 2:**
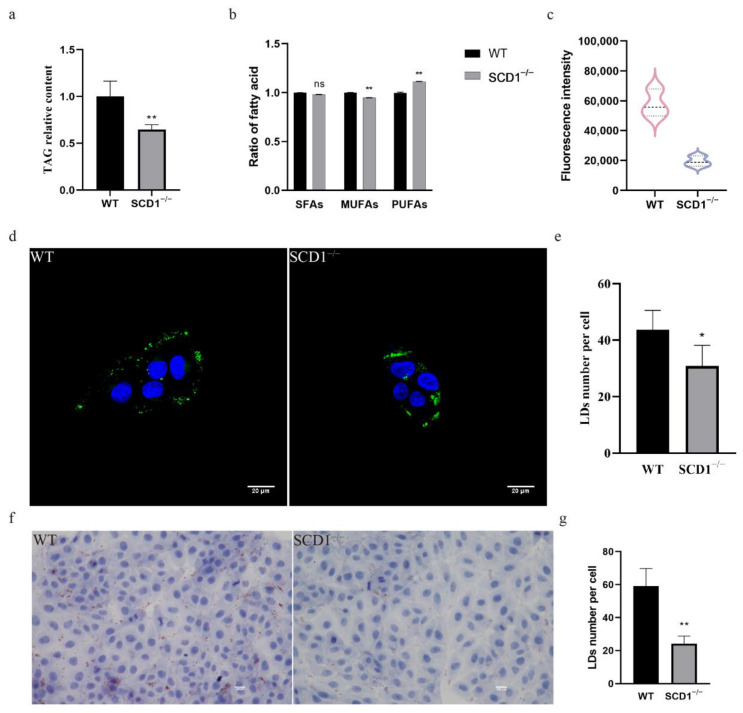
SCD1 regulates lipid metabolism and lipid droplet number: (**a**) Triglyceride level analysis; (**b**) Fatty acid content analysis; (**c**) Detection of Bodipy staining fluorescence intensity by flow cytometry; (**d**) Confocal microscopy to examine the number of lipid droplets; (**e**) imageJ calculates the number of lipid droplets in graph (**d**); (**f**) Oil Red O Staining; (**g**) imageJ calculates the number of lipid droplets in graph (**f**). * *p* < 0.05; ** *p* < 0.01; ns *p* > 0.05.

To study the effect of SCD1 deletion on fatty acid metabolism, TAG levels in SCD1-knockout cells were measured, showing a significant decrease (Figure 2a). The fatty acid content assay revealed that SCD1 deletion did not significantly affect SFA levels but reduced MUFA and increased PUFA levels in cells (Figure 2b). The absence of SCD1 reduced the ratios of the substrates palmitic acid (C16:0) and stearic acid (C18:0) to their products palmitoleic acid (C16:1) and oleic acid (C18:1) (C16:1/C16:0 and C18:1/C18:0), respectively. Additionally, SCD1 deletion decreased erucic acid (C22:1) and DHA (C22:6n3) levels and increased DPA (C22:5n3) levels, while the EPA (C20:5n3) levels were not significantly affected (Table 1). Flow cytometry analysis of Bodipy-stained SCD1^−/−^ cells showed a significant decrease in FITC fluorescence intensity compared to the control group, suggesting a significant downregulation of lipid droplet content in the SCD1-deficient group (Figure 1c). Confocal microscopy of Bodipy-stained cells showed that the number of lipid droplets was reduced in SCD1-deficient cells, as quantified by ImageJ (Figure 1d,e). Oil Red O staining further confirmed a reduction in lipid droplet numbers following SCD1 deletion (Figure 1f,g).

### 3.3. Overexpression of SCD1 Upregulates LD Number

**Figure 3:**
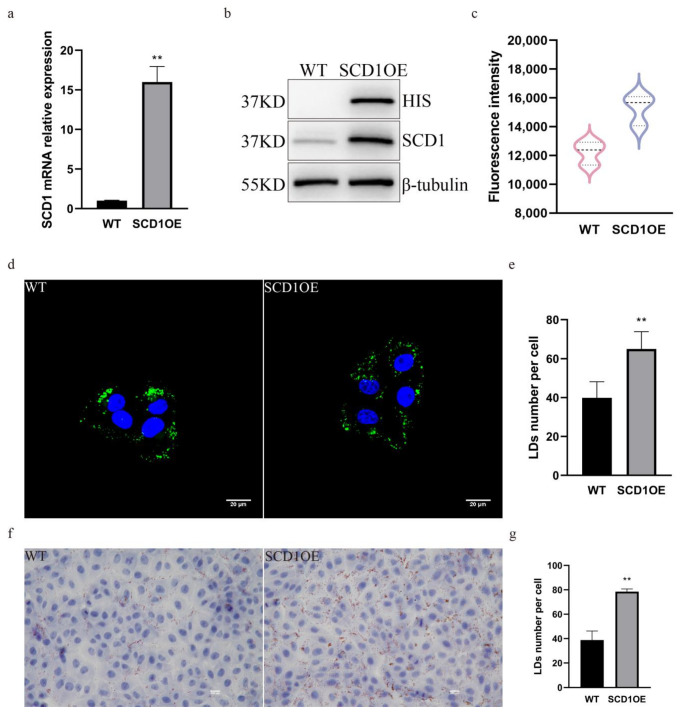
SCD1 overexpression upregulates the number of lipid droplets. (**a**) SCD1 mRNA expression level; (**b**) SCD1 protein expression level; (**c**) Detection of Bodipy staining fluorescence intensity by flow cytometry. (**d**) Confocal microscopy of BODIPY-stained cells (n = 6); (**e**) ImageJ statistics for the average number of lipid droplets per cell in Figure (**d**); (**f**) Oil Red O staining analysis; (**g**) imageJ calculates the number of lipid droplets in graph (**f**). ** *p* < 0.01.

In order to further determine the role of SCD1 in regulating the number of lipid droplets, we generated SCD1 overexpressing cells and analyzed them by PCR and WB, which showed that both the mRNA and the protein levels of SCD1 were significantly upregulated (Figure 3a,b). Bodipy staining flow cytometry was performed on SCD1 overexpression cells to detect their lipid levels, and the results showed that the average FITC fluorescence intensity of SCD1 overexpression cells was significantly upregulated, and lipid droplet content was significantly increased (Figure 3c). Cells were stained with Bodipy, imaged using a confocal microscope, and the number of lipid droplets in Figure 3d was quantified using ImageJ. The results showed that overexpression of SCD1 increased the number of lipid droplets (Figure 3d,e). The results of Oil Red O staining further indicated that the number of lipid droplets increased following SCD1 overexpression (Figure 3f,g).

### 3.4. RNA-seq Analysis Indicates That SCD1 Regulates Lipid Metabolism Through CLSTN3B

**Figure 4:**
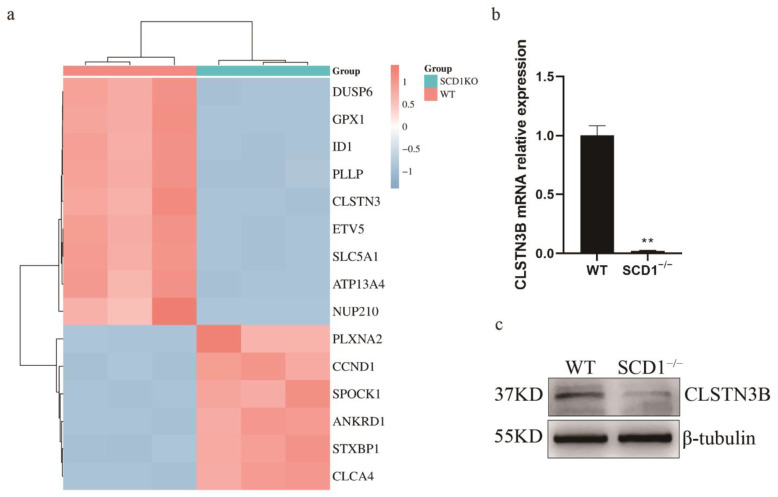
WT_vs_SCD1^−/−^ transcriptome data analysis. (**a**) WT_vs_SCD1^−/−^ top 15 Differential Gene Heatmaps; (**b**,**c**) Analysis of *CLSTN3B* mRNA and its protein expression levels in SCD1^−/−^ cells. ** *p* < 0.01.

During the analysis of differential genes, a significant downregulation of the *CLSTN3* gene was observed (Figure 4a). Its variant, *CLSTN3B*, plays a crucial role in lipid metabolism. Notably, CLSTN3B’s regulation of lipid droplet number shares a similar phenotype with SCD1. Subsequently, primers for CLSTN3B were designed following Kevin Qian’s method [16] to measure its expression levels. The results revealed a significant downregulation of *CLSTN3B* mRNA in SCD1^−/−^ cells (Figure 4b). Using a C-terminal-targeting antibody against CLSTN3, we successfully detected porcine CLSTN3B protein, which was significantly downregulated in SCD1^−/−^ cells (Figure 4c). We detected the expression level of CLSTN3B in SCD1-overexpressing cells. The results demonstrate that overexpression of SCD1 significantly affects the expression level of CLSTN3B, further supporting the regulatory role of SCD1 on CLSTN3B (Figure S1e,f).

### 3.5. SCD1 Regulates LD Number Through CLSTN3B

**Figure 5:**
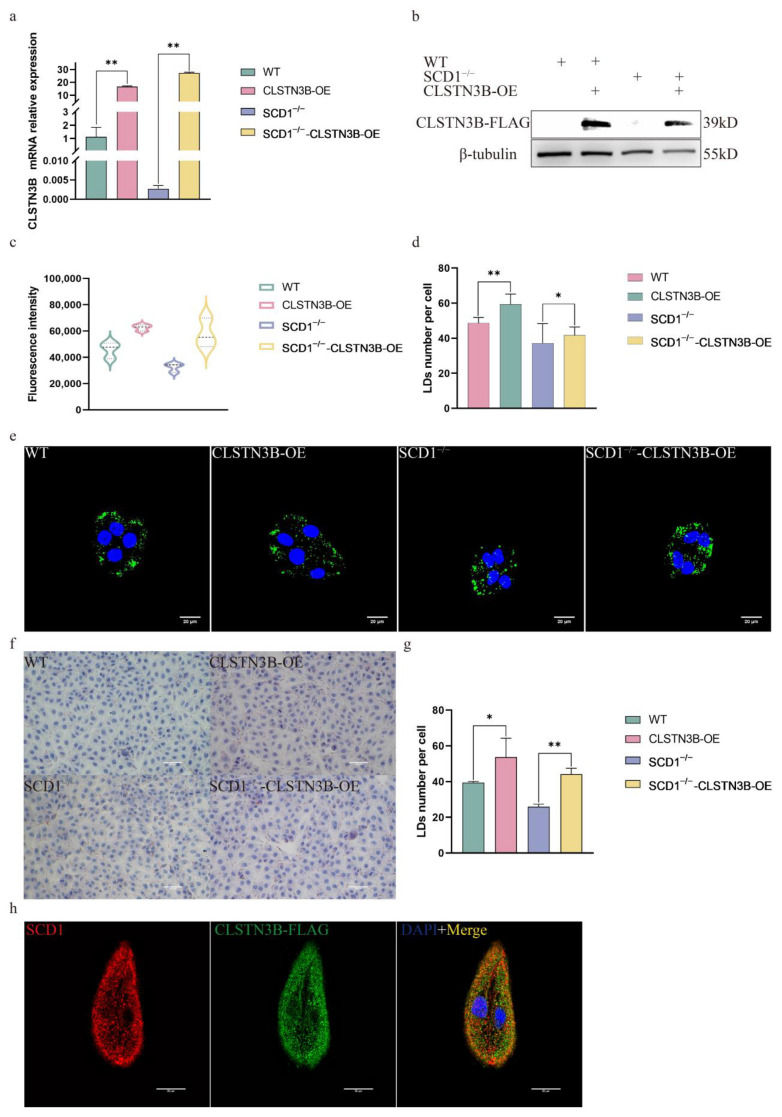
SCD1 regulates lipid droplet number through CLSTN3B (**a**,**b**) WT and SCD1^−/−^ cells transfected with CLSTN3B overexpression vector were used for qRT-PCR and Western blotting analysis; (**c**) Cells were collected for flow cytometry analysis after transfection of WT and SCD1^−/−^ cells with CLSTN3B overexpression vector; (**d**,**e**) Different groups of cells were Bodipy stained and placed under a confocal microscope for imaging, and the number of lipid droplets was analyzed using ImageJ (n = 6); (**f**) Oil Red O staining analysis; (**g**) imageJ analyzes the average number of lipid droplets in Figure (**f**); (**h**) Localization of SCD1 and CLSTN3B by immunofluorescence staining. * *p* < 0.05; ** *p* < 0.01.

To investigate whether SCD1 can regulate the lipid droplet number through CLSTN3B, we overexpressed the expression level of CLSTN3B in WT and SCD1^−/−^ cells. RT-qPCR and Western blot results showed that the expression level of CLSTN3B was successfully up-regulated in WT and SCD1^−/−^ cells (Figure 5a,b). Bodipy Staining flow cytometry assay showed that up-regulation of CLSTN3B expression level increased the average FITC fluorescence intensity of WT and SCD1^−/−^ cells, and the results indicated that up-regulation of CLSTN3B expression level increased the lipid droplet content in WT and SCD1^−/−^ cells (Figure 5c). Confocal imaging and calculation of the number of lipid droplets therein indicated that CLSTN3B overexpression upregulated the number of LD in WT and SCD1^−/−^ cells (Figure 5d,e). The Oil Red O results were consistent with those of Bodipy staining (Figure 5f,g). Further immunofluorescence staining of CLSTN3B and SCD1 showed that CLSTN3B was co-localized with SCD1 (Figure 5h).

## 4. Discussion

To explore the functional role of SCD1 in pigs, we established an SCD1-deficient PK15 cell line. Transcriptome analysis by RNA-seq revealed that the deletion of SCD1 predominantly affected pathways involved in lipid metabolism, suggesting a potential regulatory role of SCD1 in this process. To further evaluate the lipid metabolic capacity of PK15 cells, we stimulated them with oleic acid, which resulted in a marked increase in lipid droplet accumulation. These findings indicate that PK15 cells are capable of responding to lipid-related stimuli and suitable as an in vitro model for studying lipid metabolism. As a key enzyme in monounsaturated fatty acid biosynthesis, SCD1 is significantly associated with carcass traits, meat quality, fat deposition, and meat fatty acid composition [21]. SCD1 deletion decreased MUFA levels and increased PUFA levels. The ratios of the catalytic substrates palmitic acid (C16:0) and stearic acid (C18:0) to their corresponding products palmitoleic acid (C16:1) and oleic acid (C18:1) decreased with the absence of SCD1, with C16:1/C16:0 and C18:1/C18:0 ratios downregulated by 24% and 15%, respectively. Moreover, erucic acid levels decreased by 10%, whereas arachidonic acid levels increased by 18%. As a major component of lipids, an excessive amount of MUFA can affect the flavor and tenderness of meat [22]. Erucic acid is associated with mitochondrial fatty acid oxidation and hepatic steatosis with important implications for lipid metabolism [23]. The deletion of SCD1 alters fatty acid composition, suggesting that SCD1 regulates fatty acid metabolism by catalyzing the desaturation of C16:0 to C16:1 and C18:0 to C18:1, while also playing a key role in overall fatty acid homeostasis. The key characteristics of cancer development and progression are associated with the accumulation of LD, and SCD1 inhibition in CRC cells impairs TAG accumulation and lipid droplet formation [14]. SCD1 inhibition down-regulates mRNA levels of genes involved in LD formation in porcine embryos, SCD1 plays a role in regulating LD formation through phospholipid formation and embryonic development, and treatment of orphan embryos with oleic acid results in a significant increase in the rate of blastocyst formation and the number of LD [24]. The above results suggest that SCD1 plays an important role in lipid droplet biogenesis.

To elucidate the mechanism by which SCD1 regulates lipid droplet number in PK15 cells, we performed differential gene expression analysis and identified a significant downregulation of CLSTN3. CLSTN3B is an adipocyte-selective product of the *CLSTN3* locus, exhibited a similar phenotype to SCD1 in modulating lipid droplet number, which prompted further investigation. Subsequently, by designing primers specific to CLSTN3B and using a C-terminal-targeting antibody against CLSTN3, we found that both the mRNA and protein levels of CLSTN3B were significantly decreased upon SCD1 deletion. This finding further suggests that PK15 cells possess the ability to express adipocyte-specific gene products, supporting their use as a functional model for lipid metabolism studies. CLSTN3B binds to Cidea (Cell Death Inducing DFFA Like Effector A) proteins and inhibits their lipid transfer activity between LDs, thereby restricting LD fusion and growth [16]. In addition, *CLSTN3B*, through the ER-LD contact site, ultimately affects lipid droplet maturation and lipid storage by regulating the transport of phospholipids and lipids from the ER to the LD [17].

LDs are cytosolic organelles widely present in various cell types, primarily serving as storage sites for neutral lipids and regulators of cellular lipid metabolism [25]. Studies have shown that the core of LD contains TAG and sterol esters (SE), and is surrounded by a monolayer of phospholipids [26]. This phospholipid monolayer plays a critical role in regulating the morphology, structure, and function of LDs [27,28]. There are two main mechanisms through which SCD1 affects LD biogenesis. First, it alters the saturation state of inner lipids and the composition of the phospholipid monolayer [29]. During LD expansion, phospholipids from the endoplasmic reticulum (ER) must diffuse into the LD to facilitate its early growth phase [30,31]. Second, the distribution of MUFA and PUFA in the LD monolayer affects its fluidity and biophysical properties [24]. *CLSTN3B* recruits phospholipids during LD expansion to fulfill the phospholipid demands of the growing LD surface [17]. Changes in the number of LDs are closely linked to shifts in the fatty acid composition of PCs in the LD monolayer. Previous studies indicate that SCD1 plays a crucial role in porcine adipocyte differentiation, as its expression is significantly upregulated during this process, highlighting its essential function in adipogenesis [32]. Here, we show that SCD1 deficiency leads to a marked reduction in triacylglycerol (TAG) levels, alterations in fatty acid composition, and a reduction in lipid droplet number. These findings suggest that SCD1 is a potential target for modulating fat deposition in pigs. Future studies could develop SCD1-deficient animal models to further elucidate the precise mechanisms through which SCD1 modulates lipid metabolism and deposition. Additionally, investigating the interplay between SCD1 and CLSTN3B in lipid droplet biogenesis may provide valuable insights into their broader roles in adipose tissue dynamics and metabolic regulation.

The limitation of the study is that we did not use porcine preadipocytes as an in vitro model to study fat deposition. However, it would be impossible for gene knockout experiment because the limitation of passages for preadipocytes. Although PK15 cells are not adipocyte-specific, several published studies have demonstrated their suitability for investigating lipid-related processes, and their findings have been recognized by peers [33,34]. Based on the results from other research teams and our investigation, it has been shown that PK15 cells harbor functional lipid metabolism pathways, demonstrating its suitability as the cellular model for porcine lipid metabolism research.

## 5. Conclusions

This study investigated how SCD1 regulates LD numbers through its knockout and overexpression in PK15. We discovered that SCD1 influences fatty acid composition and regulates LD numbers via CLSTN3B.

## Figures and Tables

**Table 1:** Regulation of fatty acid composition by SCD1 deletion (% of total fatty acids, X ± SD, %).

Fatty Acids	WT	SCD1^−/−^
C16:0	15.74 ± 0.27	20.65 ** ± 0.22
C16:1	3.23 ± 0.03	3.23 ^ns^ ± 0.02
C18:0	23.35 ± 0.30	20.85 ** ± 0.21
C18:1n9c	36.72 ± 0.49	28.17 ** ± 0.29
C22:1n9	0.55 ± 0.01	0.56 ^ns^ ± 0.01
C24:1	0.45 ± 0.02	0.29 ** ± 0.01
C18:2n6t	0.045 ± 0.01	0.05 * ± 0.01
C18:2n6c	5.78 ± 0.05	7.80 ** ± 0.04
C18:3n6	0.13 ± 0.01	0.27 ** ± 0.01
C18:3n3	0.10 ± 0.01	0.18 ** ± 0.01
C20:4n6	11.36 ± 0.14	15.23 ** ± 0.18
C20:5n3	0.34 ± 0.01	0.40 ** ± 0.01
C22.5n6	0.70 ± 0.01	0.77 * ± 0.01
C22.6n3	1.50 ± 0.01	1.52 ^ns^ ± 0.01

* *p* < 0.05; ** *p* < 0.01; ^ns^ *p* > 0.05.

## Data Availability

Raw data can be obtained by contacting the corresponding author.

## Supplementary Materials

The following supporting information can be downloaded at: https://www.mdpi.com/article/10.3390/ani15111663/s1, Table S1: Primer sequences used and sgRNA oligonucleotide sequences. Figure S1a: Sanger sequencing results showing that the sgRNA induced a 4 bp (AGAC) deletion in the first exon of the SCD1 gene; Figure S1b: Schematic diagram of the frameshift mutation caused by the AGAC deletion (red text indicates the deletion site, yellow indicates the premature stop codon, and blue represents the sequences flanking the deletion site); Figure S1c: SCD1 mRNA expression levels; ns indicates no significant difference (*p* > 0.05); Figure S1d: SCD1 protein expression levels; Figure S1e,f: Analysis of CLSTN3B mRNA and protein expression levels in SCD1-overexpressing (SCD1-OE) cells; Figure S2a: Using the online analysis tool Cas-OFFinder (http://www.rgenome.net/cas-offinder/ accessed on 28 May 2025), potential off-target sites of the sgRNA in the pig genome were analyzed. As shown in the figure, there were no off-target sites with 1 or 2 mismatches. There were 2 potential off-target sites with 3 mismatches and a total of 54 sites with 4 mismatches; Figure S2b: Mismatch analysis between the sgRNA and potential off-target sites with 3 mismatches (Note: Sequence 0 represents the original sgRNA sequence and its genomic location; matched bases are highlighted in green, mismatched positions are shown in white, and the PAM sequence is marked in red); Figure S2c: Mismatch analysis between the sgRNA and potential off-target sites with 4 mismatches; Figure S2d: PCR analysis of potential off-target sites; Figure S3a–o: Sequencing analysis of potential off-target sites.
