# Supplementary material for: Porcine SCD1 Regulates Lipid Droplet Number via CLSTN3B in PK15 Cells
Source: Animals (Basel). 2025 Jun 4;15(11):1663. doi: 10.3390/ani15111663 (PMC12153863; doi:10.3390/ani15111663)
Supplement: Supplementary file 1 [file animals-15-01663-s001.zip › animals-3624122-supplementary.pdf]

Supplementary Materials:

Table S1. Primer sequences used and sgRNA oligonucleotide sequences

Gene	Accession	Primer Sequences (5′ to 3′)	Product Size (bp)
SCD1	NM_213781.1	F：GCCACCTTTCTTCGTTACG<br>R：CCTCACCCACAGCTCCCAAT	142
CLSTN3B	XM_021092406.1	F：CTTCTGGGACGACTCAGCTC<br>R：TGATGCGTCTCTCGTCACTG	161
GAPDH	NM_001206359.1	F：TCGGAGTGAACGGATTTGGC<br>R：TGACAAGCTTCCCGTTCTCC	189
pcDNA3.1-SCD1-6*HIS	NM_213781.1	F：tagtccagtgtggtggaattCATGCCGGCCCACTTGCTG<br>R：tcagcgggtttaaacgggCCCTCAGGCACTGCCGTCTCCA	1080
sgRNA		AAGTAATGGCCCCCAGACCGCGG	

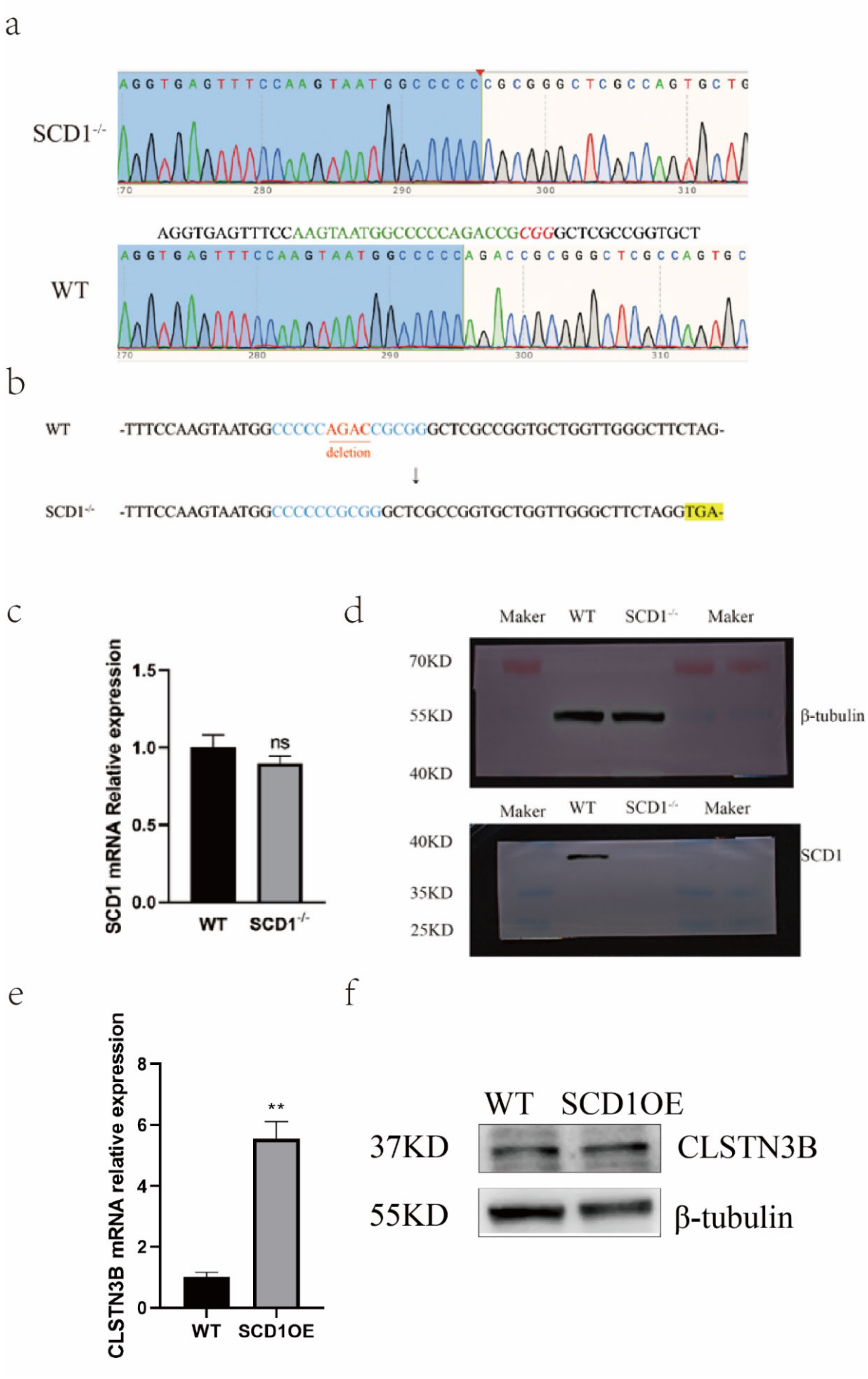

Figure S1. (a) Sanger sequencing results showing that the sgRNA induced a 4 bp (AGAC) deletion in the first exon of the SCD1 gene; (b) Schematic diagram of the frameshift mutation caused by the AGAC deletion (red text indicates the deletion site, yellow indicates the

premature stop codon, and blue represents the sequences flanking the deletion site); (c) SCD1 mRNA expression levels; ns indicates no significant difference ($p > 0.05$); (d) SCD1 protein expression levels; (e–f) Analysis of CLSTN3B mRNA and protein expression levels in SCD1-overexpressing (SCD1-OE) cells. * $p < 0.05$; ** $p < 0.01$.

a

Summary

Target Sequence	Bulge Type	Bulge Size	Mismatch	Number of Found Targets
AAGTAATGGCCCCCAGACCGNGG	X	0	0	1
AAGTAATGGCCCCCAGACCGNGG	X	0	3	2
AAGTAATGGCCCCCAGACCGNGG	X	0	4	54

b

Name	Location	1	2	3	4	5	6	7	8	9	10	11	12	13	14	15	16	17	18	19	20	P	A	M
0		A	A	G	T	A	A	T	G	G	C	C	C	C	C	A	G	A	C	C	G	G	G	G
3-1	CHR8-58467786	A	A	a	T	A	A	T	G	G	C	C	C	C	t	A	G	A	C	C	c	T	G	G
3-2	CHR15-96556925	A	A	G	T	A	A	T	t	G	C	C	C	C	a	A	G	A	C	C	c	T	G	G

c

Name	Location	1	2	3	4	5	6	7	8	9	10	11	12	13	14	15	16	17	18	19	20	P	A	M
0		A	A	G	T	A	A	T	G	G	C	C	C	C	C	A	G	A	C	C	G	G	G	G
4-1	90782481	A	A	G	c	A	A	T	G	c	C	C	C	C	C	A	c	A	C	C	c	A	G	G
4-2	CHR2-70091176	A	A	G	T	A	A	a	G	G	C	C	a	C	C	A	G	A	C	a	c	T	G	G
4-3	CHR3-15452476	A	A	G	T	A	A	T	G	c	a	C	C	C	C	A	G	A	a	t	G	G	G	G
4-4	CHR3-28447612	g	A	G	T	A	A	a	G	G	C	C	C	g	C	A	t	A	C	C	G	G	G	G
4-5	CHR3-30355624	A	g	G	c	A	A	T	G	G	C	C	C	C	C	A	G	A	t	C	c	T	G	G
4-6	CHR475838432	A	A	G	T	A	A	T	t	G	C	C	C	C	a	A	G	A	C	C	c	T	G	G
4-7	CHR4-77793137	A	A	G	T	g	t	T	G	G	C	C	C	a	C	A	G	A	t	C	G	T	G	G
4-8	CHR4-105116930	A	A	G	g	A	A	T	G	G	C	C	C	a	C	g	G	A	C	C	c	T	G	G
4-9	CHR3-32218084	A	A	G	T	A	A	T	G	G	C	C	C	C	C	A	G	A	C	C	G	C	G	G
4-10	CHR5-2010347	A	A	c	T	c	c	a	G	G	C	C	C	C	C	A	G	A	C	C	G	A	G	G
4-11	CHR6-52210578	c	A	G	g	A	A	T	G	G	a	a	C	C	C	A	G	A	C	C	G	A	G	G
4-12	CHR1-111462020	A	A	G	T	A	A	T	G	G	C	C	C	C	C	A	G	A	C	C	G	C	G	G
4-13	CHR17-48176070	c	A	G	g	A	A	T	G	G	a	a	C	C	C	A	G	A	C	C	G	A	G	G

d

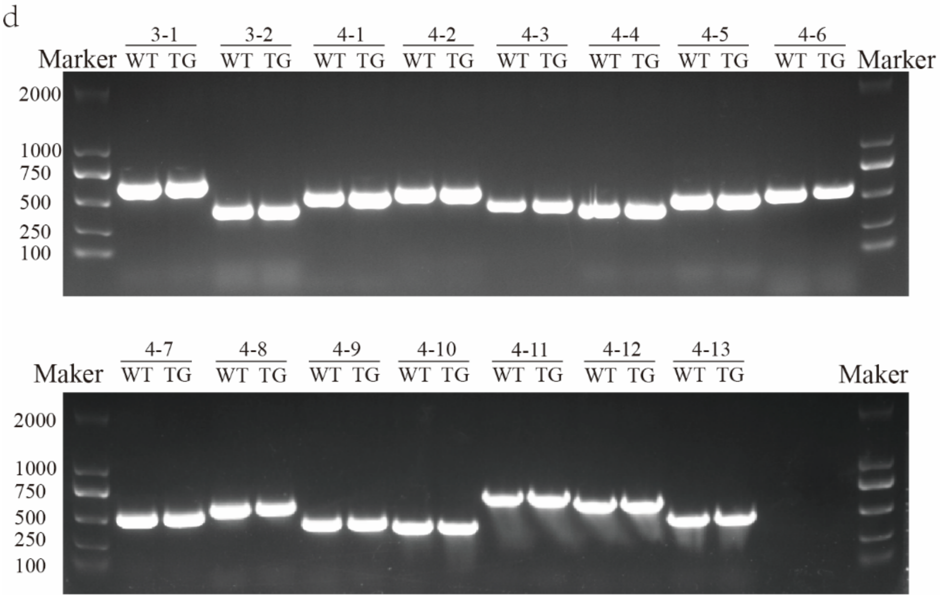

Figure S2. Analysis of potential off-target sites by Cas-OFFinder. (a) Using the online analysis tool Cas-OFFinder (http://www.rgenome.net/cas-offinder/), potential off-target sites of the sgRNA in the pig genome were analyzed. As shown in the figure, there were no off-target sites with 1 or 2 mismatches. There were 2 potential off-target sites with 3 mismatches and a total of 54 sites with 4 mismatches; (b) Mismatch analysis between the sgRNA and potential off-target sites with 3 mismatches (Note: Sequence 0 represents the original sgRNA sequence and its genomic location; matched bases are highlighted in green, mismatched positions are shown in white, and the PAM sequence is marked in red); (c) Mismatch analysis

between the sgRNA and potential off-target sites with 4 mismatches (Note: Sequence 0 represents the original sgRNA sequence and its genomic location; matched bases are highlighted in green, mismatched positions are shown in white, and the PAM sequence is marked in red); (d) PCR analysis of potential off-target sites.

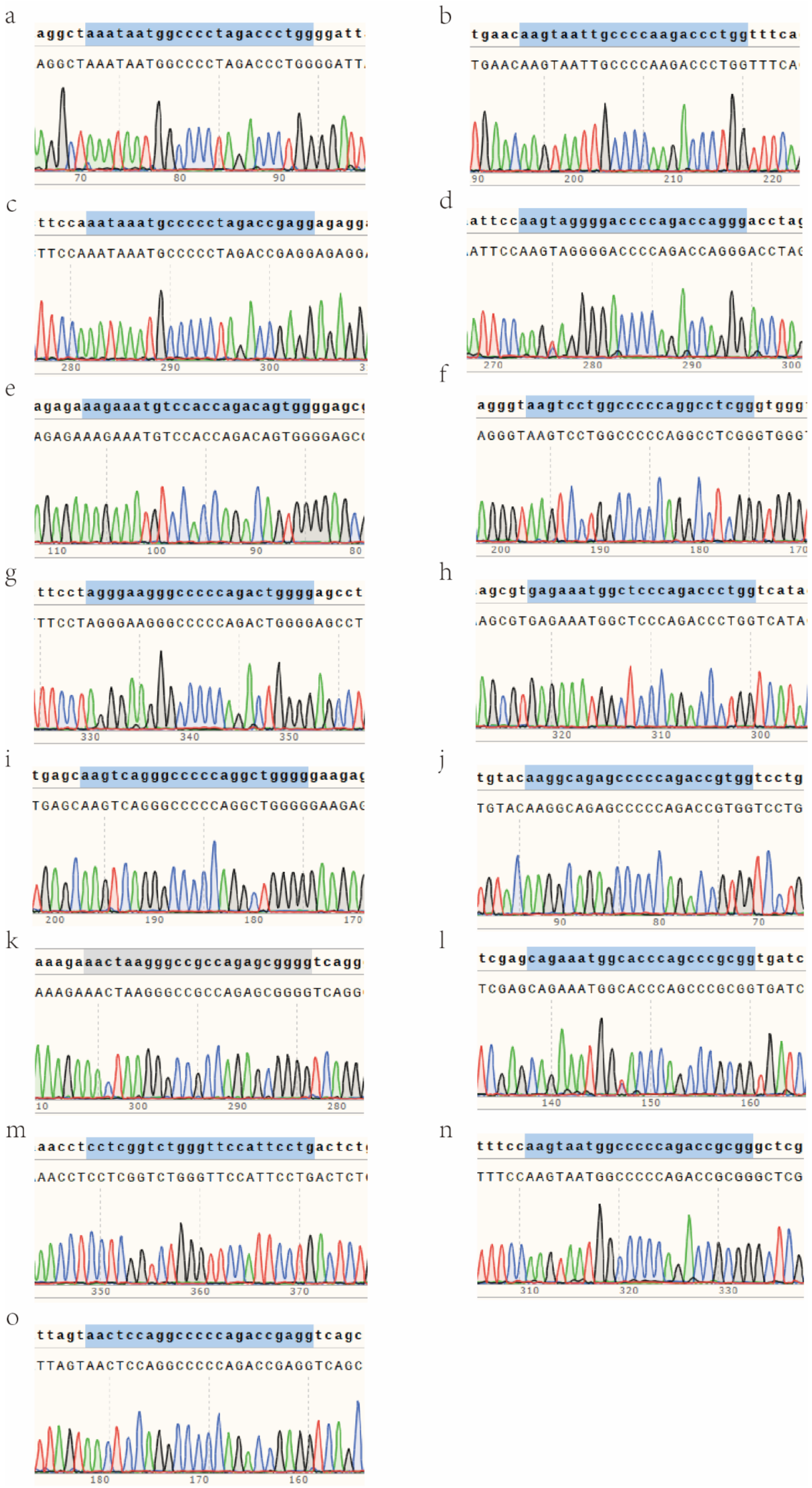

Figure S3. Sequencing analysis of potential off-target sites. Potential off-target sites are shown in blue, with the Sanger sequencing results displayed below. (a) Sequencing results of potential off-target site 3-1; (b) Sequencing results of potential off-target site 3-2; (c) Sequencing results of

potential off-target site 4-1; (d) Sequencing results of potential off-target site 4-2; (e) Sequencing results of potential off-target site 4-3; (f) Sequencing results of potential off-target site 4-4; (g) Sequencing results of potential off-target site 4-5; (h) Sequencing results of potential off-target site 4-6. (i) Sequencing results of potential off-target site 4-7; (j) Sequencing results of potential off-target site 4-8; (k) Sequencing results of potential off-target site 4-9; (l) Sequencing results of potential off-target site 4-10; (m) Sequencing results of potential off-target site 4-11; (n) Sequencing results of potential off-target site 4-12; (o) Sequencing results of potential off-target site 4-13.
